# Iron-oxidizing microorganisms affect the iron-bound organic carbon in the subsoil of alpine grassland during the thawing of seasonal frozen soil

**DOI:** 10.3389/fmicb.2024.1523084

**Published:** 2025-01-06

**Authors:** Yuxin Tian, Maidinuer Abulaizi, Zailei Yang, Tianle Kou, Yuanbin Jia, Yunpeng Hu, Mo Chen, Hongtao Jia

**Affiliations:** ^1^College of Grassland Science, Xinjiang Agricultural University, Urumqi, China; ^2^College of Resources and Environment, Xinjiang Agricultural University, Urumqi, China; ^3^Xinjiang Key Laboratory of Soil and Plant Ecological Processes, Xinjiang Agricultural University, Urumqi, China

**Keywords:** alpine grassland, thawing of seasonal frozen soil, Fe-cycling microorganisms, Fe-bound organic carbon, Fe-cycling functional genes

## Abstract

Iron (Fe) minerals possess a huge specific surface area and high adsorption affinity, usually considered as “rust tanks” of organic carbon (OC), playing an important role in global carbon storage. Microorganisms can change the chemical form of Fe by producing Fe-chelating agents such as side chains and form a stable complex with Fe(III), which makes it easier for microorganisms to use. However, in seasonal frozen soil thawing, the succession of soil Fe-cycling microbial communities and their coupling relationship with Fe oxides and Fe-bound organic carbon (Fe-OC) remains unclear. We characterized changes in the Fe phase, Fe-OC, Fe-oxidizing bacteria (FeOB), and Fe-reducing bacteria (FeRB) in the subsoil and analyzed the microbial mechanism underlying Fe-OC changes in alpine grassland by constructing a composite structural equation model (SEM). We found that the Fe(III) content consistently exceeded that of Fe(II). Among the three types of Fe oxides, organically complex Fe (Fe_p_) decreased from 2.54 to 2.30 g·kg^−1^, whereas the opposite trend was observed for poorly crystalline Fe (Fe_o_). The Fe-OC content also decreased (from 10.31 to 9.47 g·kg^−1^; *p* < 0.05). Fe-cycling microorganisms were markedly affected by the thawing of frozen soil (except FeRB). Fe_p_ and Fe_o_ directly affected changes in Fe-OC. Soil moisture (SM) and FeOB were significant indirect factors affecting Fe-OC changes. Freeze–thaw changes in the subsoil of alpine grassland in Central Asia significantly affected FeOB and Fe oxides, thus affecting the Fe-OC content. To the best of our knowledge, this was the first study to examine the influence of Fe-cycling microorganisms on the Fe phase and Fe-OC in the soil of alpine grassland in Central Asia. Overall, our findings provide scientific clues for exploring the biogeochemical cycle process in future climate change.

## 1 Introduction

One-third of the global soil C is stored in permafrost soil. As an important C sink, permafrost soil plays a vital role in the global C cycle (Marushchak et al., 2021). The storage and stability of soil organic carbon (SOC) in permafrost regions are critically important for the feedback between the terrestrial C cycle and the warming of the climate and have attracted increased research attention (Song et al., 2019; Wild et al., 2019; Chen et al., 2024). However, seasonal permafrost is also widespread, and it comprises approximately 51% of the total land area in the Northern Hemisphere (Zhang et al., 2003). Seasonal frozen soil is defined as near-subsoil that freezes for more than 15 days every year; the active layer in the permafrost region also belongs to seasonal frozen soil (Zhang et al., 2003). The hydrological cycle is more active in seasonal frozen regions than in permafrost regions, and its effect on SOC transformation and output is more significant in the former than in the latter (Zhang et al., 2023). Under the background of global warming, the maximum frozen soil depth is becoming shallower, the freezing period is shortening, the thawing period is becoming longer, and the range of frozen soil is shrinking, which has a major effect on groundwater circulation, C cycle, and even regional sustainable development (McCalley et al., 2014; Olid et al., 2020; Luo et al., 2020).

With the thawing of frozen soil, the previously frozen SOC can be used for mineralization (Zimov et al., 2006), which leads to the release and export of C (Mu et al., 2015; Turetsky et al., 2020). Therefore, the study of SOC stability is particularly important. The stability of SOC depends on its complex interactions with minerals (Singh et al., 2018). Fe minerals can be used as an effective “rust sink” to capture terrestrial SOC in sediments, which is essential for the long-term storage of SOC and makes an important contribution to the global C cycle (Song et al., 2022). The stocks of active Fe minerals are highly dynamic, and they are actively precipitated or dissolved in response to changing redox conditions (Patzner et al., 2020). Fe has a protective effect on SOC under static aerobic conditions; under oxygen-limited conditions, it leads to the anaerobic mineralization of SOC (Chen et al., 2020). The Fe cycle is an important biogeochemical process, and the microbial Fe cycle is a major driver of biogeochemical cycles of other elements (Liu Y. et al., 2022).

Fe-cycling microorganisms play a dual role in SOC mineralization as Fe is not only the terminal electron acceptor of SOC mineralization but also the basis of chemical protection after SOC binding (Liu et al., 2019). Fe minerals can be used as SOC adsorbents and contribute to SOC protection by providing structural support, and the dissimilatory Fe reduction process in *Shewanella oneidensis MR-1* is capable of liberating C from its bound form (Pan et al., 2016). FeOB and FeRB profoundly influence the conversion of Fe oxides and the mineralization process of SOC (Bi et al., 2024). Sutton-Grier et al. (2011) quantified the contribution of OC mineralization in soil with wetland plants. The reduction of Fe played a pivotal role in the mineralization of OC, with microorganisms accounting for a significant 65% of the Fe-reducing process (Sutton-Grier et al., 2011). Fe oxidation is mainly regulated by the *iro* gene, and Fe reduction is mainly regulated by the *OmcS* gene; these two genes are significantly related to the degradation of unstable OC (Liu S. et al., 2022). Nevertheless, the structural dynamics and functional gene expression within the microbial community involved in the Fe cycle remain unexplored during the thawing of frozen soil. Therefore, the study of the relationship between Fe and C requires consideration of the microbial-mediated redox process of the Fe cycle, which has a major effect on the transformation and storage of C in the soil environment (Duan et al., 2020).

Since the permafrost undergoes a top-down thawing process and the surface soil responds first to environmental changes, most of the current research focuses on surface soil (Tedersoo et al., 2014; Thompson et al., 2017; Dove et al., 2021; Xu et al., 2024). In contrast, the subsoil is less disturbed by external environmental factors and can provide relatively stable living conditions for microorganisms (Mosley et al., 2022). In addition, the migration and transformation of water in the subsoil during the thawing of frozen soil are more complicated (Li et al., 2022). Therefore, it is important to investigate the reaction of the Fe phase and microbial community within subsoil to alterations in the hydrothermal environment to gain a comprehensive understanding of the regulatory mechanism governing the changes in Fe-OC during the thawing of seasonal frozen soil. Bayinbuluk alpine grassland experiences a typical alpine climate and is highly sensitive to global climate change. The seasonal freezing and thawing period can last up to 6 months (Chen et al., 2021; Chen et al., 2023). We studied the seasonal thawing process of the subsoil in Bayinbuluk alpine grassland; explored changes in the Fe phase, Fe-OC, and microbial community over time using metagenome sequencing technology; and analyzed the microbial mechanism of Fe-OC changes in alpine grassland during the thawing of frozen soil. We proposed three hypotheses: (1) The Fe-OC content in the subsoil decreases significantly during the thawing of frozen soil; (2) FeRB is more affected by the thawing of frozen soil than FeOB; and (3) the Fe-OC content is significantly affected by FeRB and the Fe phase.

## 2 Materials and methods

### 2.1 Overview of the study area

Bayinbuluk alpine grassland (42°18′ ~ 43°34′N, 82°27′ ~ 86°17′E) is the second largest alpine grassland in China. It is located in the inner section of Hejing County, Xinjiang Uygur Autonomous Region, China. Bayinbuluk alpine grassland experiences special climatic conditions and has a unique geographical location; this grassland is highly sensitive to global climate change. The average annual rainfall in the study area is 273 mm, the average annual temperature is −4.8°C, the lowest temperature recorded is −49.6°C, the freezing and thawing period is as long as 6 months, and the historical maximum frozen soil depth is 4.39 m (the highest value recorded by the China Meteorological Station; Chen et al., 2023; Hu et al., 2023). Based on the long-term monitoring transect, historical soil environment data, and real-time data, we focused our studies on soil in the swamp meadow area. The soil type of the study area is alpine meadow soil.

### 2.2 Sample collection

In the experimental area, five experimental plots with flat terrain and consistent vegetation growth were randomly selected. Weather stations (U30-NRC, HOBO, USA) and TDR (150, SPECTRUM, USA) probes were used to collect data on soil temperature (ST) and soil moisture (SM). Based on historical records and real-time ST and SM data, three sampling time nodes were designed during the thawing of frozen soil in 2023: deep freezing period M2 (early February), initial thawing period M4 (early April), and complete thawing period M6 (early June; Chen et al., 2021).

When sampling, the litter was removed, and random subsoil samples (20–40 cm) were collected in plots via the five-point sampling method. To avoid metal interference, the six surfaces of the soil samples were evenly cut off by 1–2 cm using a ceramic knife. Some samples were stored at 4°C for Fe and Fe(II) determination. Some samples were stored in liquid nitrogen tanks for metagenome sequencing. Some samples were brought to the laboratory and air-dried naturally; some samples were used to determine Fe oxide-related indexes, while others were used to determine the content of Fe-OC.

### 2.3 Determination indicators

#### 2.3.1 Determination of Fe oxides

The extraction of Fe_d_ (free Fe oxides), Fe_o_ (poorly crystalline Fe oxides), and Fe_p_ (organically complexed Fe oxides) was carried out using dithionite–citrate–bicarbonate (DCB) solution, ammonium oxalate–oxalic acid solution, and sodium pyrophosphate solution, respectively (Weiss et al., 2003). Then, the Fe content was determined by flame atomic absorption spectrometry (Agilent Technologies Inc., USA).

#### 2.3.2 Determination of Fe

Fe(III) and Fe(II) extracted using 0.5 M hydrochloric acid (HCl) were dissolved into Fe substances. After adding the reducing agent (hydroxylamine hydrochloride), the total Fe extracted by HCl was determined. Fe(III) extracted by hydrochloric acid was calculated as the difference between total Fe and Fe(II) extracted by hydrochloric acid (Kostka and Luther III, 1994). Fe was determined using UV spectrophotometry (Shimazu UV-2500, Japan) via 1,10-phenanthroline colorimetry to prevent the accidental oxidation of Fe(II) (Zou et al., 2009).

#### 2.3.3 Determination of Fe-OC

The control soil samples were extracted using sodium chloride (NaCl) solution with an ionic strength equivalent to that of DCB solution. OC in soil residue was extracted and its content was determined using an elemental analyzer (Lalonde et al., 2012). The formula for calculating Fe-OC was as follows:


$$Fe-OC={OC}_{\text{NaCl}}-{OC}_{DCB}$$

Where OC_NaCl_ and OC_DCB_ are the OC contents in soil residue after the extraction of sodium chloride and dithionite–citrate–bicarbonate, respectively.

#### 2.3.4 Metagenome sequencing analysis

Trimmomatic (v. 0.33) was applied to refine the raw data, yielding superior sequencing data (clean tags). MEGAHIT (v. 1.1.2) was used, with sequences of less than 300 bp being excluded. QUAST (v. 2.3) was then implemented to assess the quality of the assembly outcomes. MetaGeneMark (v. 3.26) was used to pinpoint the coding sequences within the genome. MMseq2 (v. 11-e1a1c) serves to eliminate redundancy, with a similarity cutoff of 95% and a coverage threshold set at 90%. By integrating the functional gene set related to the element cycle in KEGG, Metacyc, CAZy, KOFAM, Pfam, TIGRfam, dbCan2, MEROPs, Mcyc, Ncyc, Pcyc, Scyc, and other databases, the functional gene set of the PSN biogeochemical cycle was created, and the annotation of functional genes related to the Fe cycle was completed (Shanghai Personal Biotechnology Co., Ltd.). All raw sequence data have been submitted to the NCBI Sequence Read Archive under the BioProject identifier: PRJNA1180693.

### 2.4 Statistical analysis

Complete data analysis and drawing were performed R (v.4.1.1; https://www.r-project.org/) and ggplot2 (v.3.4.4) package. The EasyStat (v.0.1.0) package was used to test the normality, homogeneity of variance, and the differences between groups of soil indexes (temperature, moisture, and Fe phase). The vegan (v.2.5-7) package was used for non-metric multidimensional scaling (NMDS) analysis, permutational multivariate analysis of variance (PERMANOVA), analysis of similarities (ANOSIM), and beta displaced test analysis based on Fe-cycling genera and genes. The microeco (v.0.12.1) package and pheatmap (v.1.0.12) package were used to make a heat map of the abundance changes of Fe-cycling genera and genes. The LinkET (v.0.0.1.1) package was used for correlation analysis and mantel test between soil indexes and Fe-cycling genera and genes, respectively. The ggvegan (v.0.1.0) package and vegan (v.2.5-7) package were used for redundancy analysis (RDA) to show the importance of the changes of soil indexes to the changes of Fe-cycling genera and genes. Based on the influence of soil moisture on Fe-cycling genera, genes, and Fe phase, TpiecewiseSEM (v.2.1.2) package was used to model the composite structural equation, and Fisher’s C-test (0.05 < *p* < 1.00) was conducted to confirm the modeling goodness (Tian et al., 2021; Chen et al., 2023).

## 3 Results

### 3.1 Effects of seasonal frozen soil thawing on ST, SM, and Fe phase

**Figure 1:**
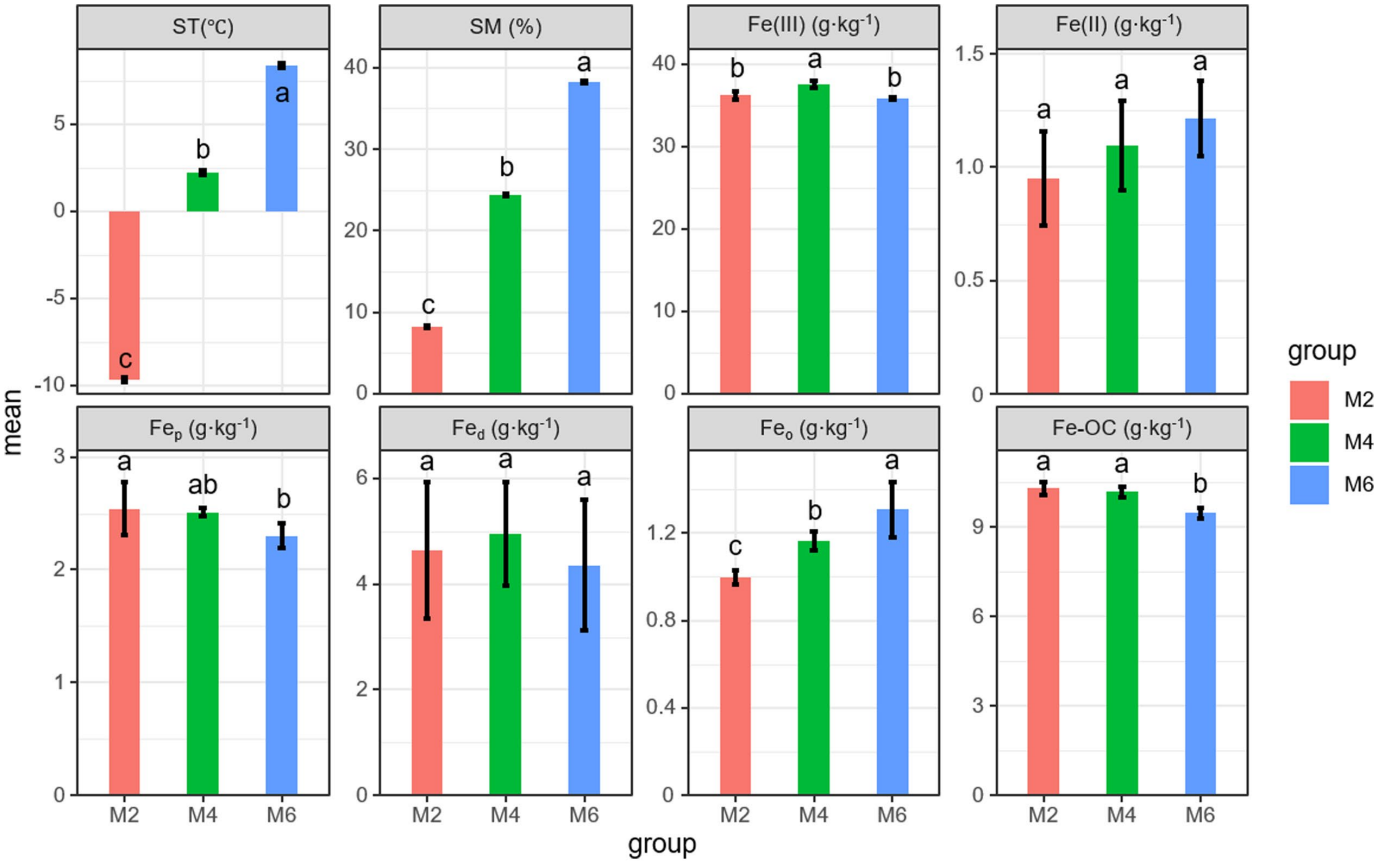
Effects of seasonal frozen soil thawing on ST, SM, and Fe phase. Different letters indicate significant differences between groups at the 0.05 level. ST, soil temperature; SM, soil moisture; Fe_d_, free Fe oxides; Fe_o_, poorly crystalline Fe oxides; Fe_p_, organically complexed Fe oxides; Fe-OC, Fe-bound organic carbon.; M2, deep freezing period (early February); M4, initial thawing period (early April); M6, complete thawing period (early June).

ST and SM increased during the thawing of frozen soil (Figure 1). The content of Fe(III) in soil was always higher than that of Fe(II). The content of Fe(III) changed significantly, and it is highest at the M4 stage with a content of 37.56 g·kg^−1^ (*p* < 0.05). The content of Fe(II) increased, but there were no significant differences (*p* > 0.05). Fe_p_ significantly decreased during the thawing of seasonal frozen soil (*p* < 0.05). However, the opposite pattern was observed for Fe_o_ (*p* < 0.05). The content of Fe_o_ was the lowest among the three types of Fe oxides, and the content of Fe_d_ first increased and then decreased. No significant differences in the Fe_d_ content were observed among stages (*p* > 0.05). The content of Fe-OC significantly decreased. These results indicated that with the thawing of seasonal frozen soil, the content of different forms of Fe in soil changed, resulting in a gradual decrease in the content of Fe-OC in soil.

### 3.2 Changes of community composition in Fe-cycling microorganisms and correlation analysis

**Figure 2:**
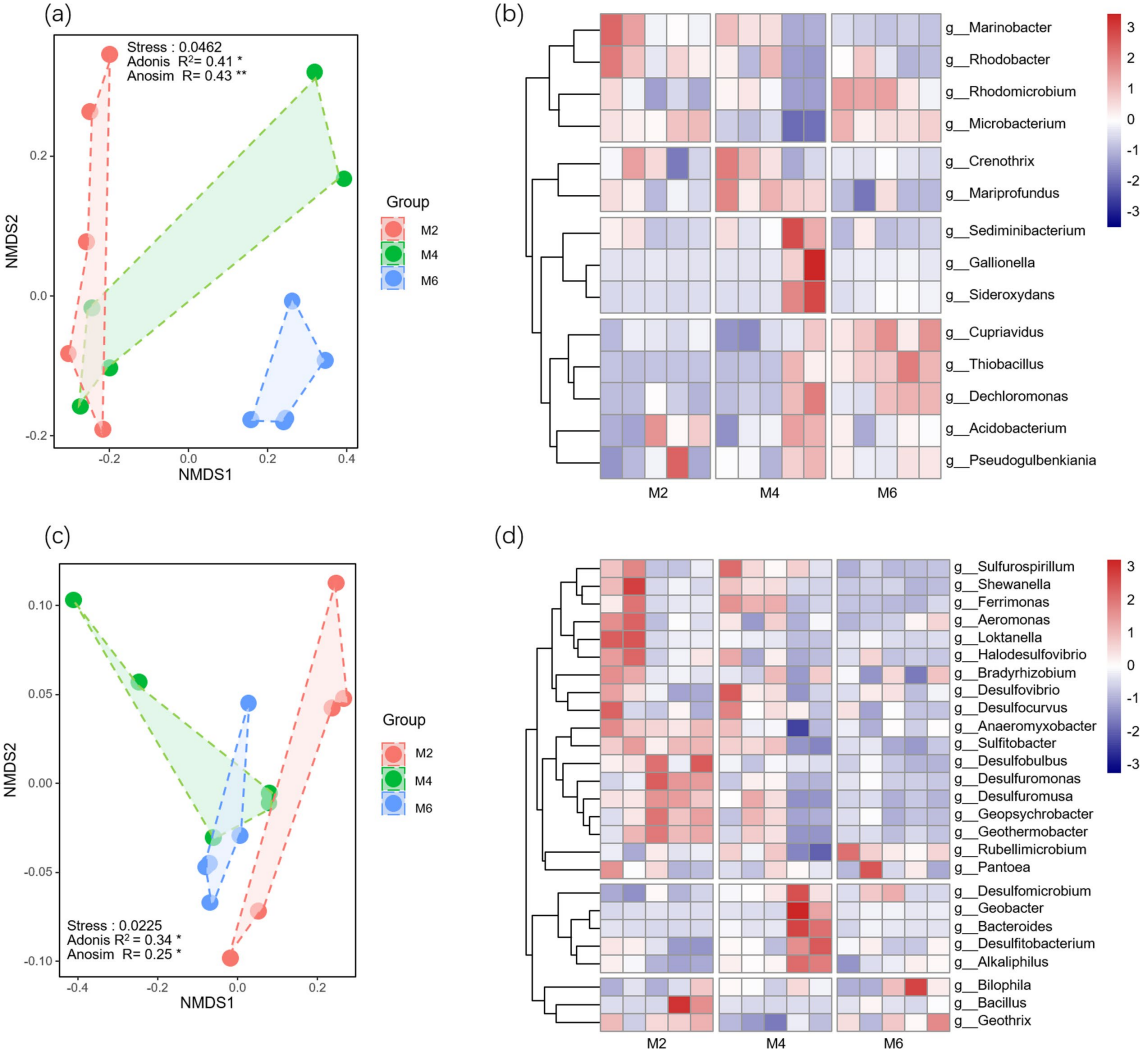
**(a)**Temporal variation characteristics of β diversity in FeOB community; **(b)** Temporal variation characteristics of FeOB community composition;**(c)** Temporal variation characteristics of β diversity in FeRB community; **(d)** Temporal variation characteristics of FeRB community composition. * and ** indicate significance at the 0.05 and 0.01 levels, respectively. The points in NMDS represent samples, and different colors represent the information of the group to which the samples belong. The distance between points in the same group indicates the degree of dispersion of samples. M2, deep freezing period (early February); M4, initial thawing period (early April); M6, complete thawing period (early June).

Adonis substitution multivariate analysis of variance (R^2^ = 0.41, *p* < 0.01) and ANOSIM similarity analysis (R = 0.43, *p* < 0.01) revealed significant differences among the FeOB groups (Figure 2A). Adonis substitution multivariate analysis of variance (R^2^ = 0.34, *p* < 0.01) and ANOSIM similarity analysis (R = 0.25, *p* < 0.01) revealed significant differences among the FeRB groups (Figure 2C). Clear clustering of FeOB was observed at the genus level (Figure 2B). Subcommunity 3 (Sub 3) contained three genera: *g_Sedinibacterium*, *g_Gallionella*, and *g_Sideroxydans*. These genera were higher in the M4 group. However, five genera in sub 4 were higher in the M6 group. Marked clustering of FeRB was also observed (Figure 2D). Sub 1 contained 18 genera was higher in the M2 and M4 groups. Sub2 contained five genera, such as *g_Desulfofobium* and *g_Geobacter*, which was higher in the M4 group.

**Figure 3:**
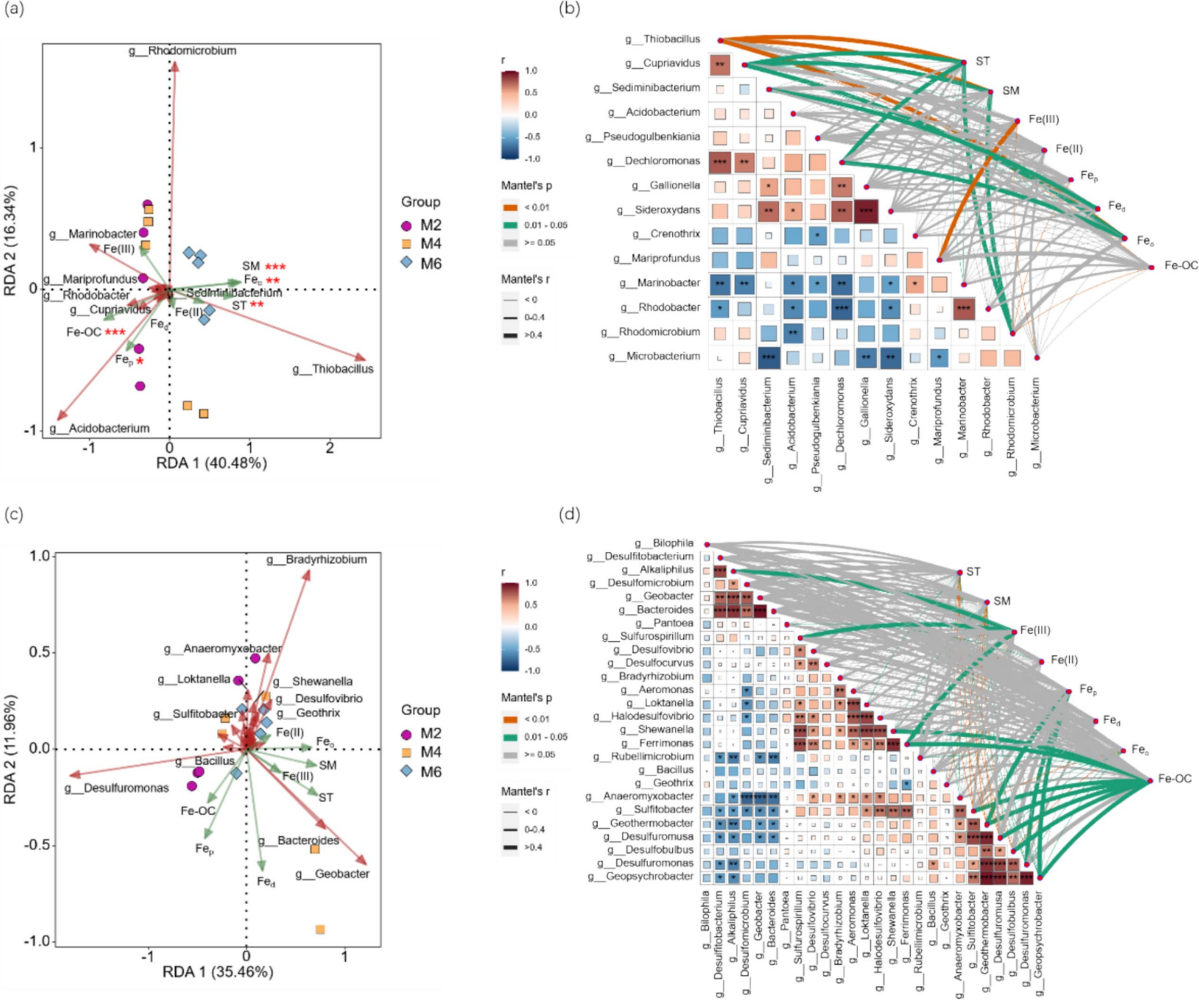
**(a)** RDA analysis was performed with FeOB community as the response variable and ST, SM, Fe(III), Fe(II), Fe_d_, Fe_o_, Fe_p_, and Fe-OC as the explanatory variables; **(b)** Correlation analysis between FeOB community and examining factors; **(c)** RDA analysis was performed with FeRB community as the response variable and ST, SM, Fe(III), Fe(II), Fe_d_, Fe_o_, Fe_p_, and Fe-OC as the explanatory variables; **(d)** Correlation analysis between FeRB community and examining factors. *, **, and *** indicate significance at the 0.05, 0.01, and 0.001 levels, respectively. ST, soil temperature; SM, soil moisture; Fe_d_, free Fe oxides; Fe_o_, poorly crystalline Fe oxides; Fe_p_, organically complexed Fe oxides; Fe-OC, Fe-bound organic carbon; M2, deep freezing period (early February); M4, initial thawing period (early April); M6, complete thawing period (early June).

RDA analysis was performed with the FeOB or FeRB community as the response variable and ST, SM, Fe(III), Fe(II), Fe_d_, Fe_o_, Fe_p_, and Fe-OC as the explanatory variables. The FeOB community was significantly affected by ST, SM, Fe_p_, Fe_o_, and Fe-OC (*p* < 0.05; Figure 3A). The FeRB community was not affected by any of the factors examined (Figure 3C). Correlation analysis of FeOB at the genus level (Figure 3B) and examining factors revealed that Fe_o_, ST, and SM were extremely significantly correlated with *g_Thiobacillus* (*p* < 0.01). Fe(III) was positively correlated with *g_Mariprofundus* (*p* < 0.01). Fe-OC was negatively correlated with *g_Thiobacillus*, *g_Cupriavidus*, and *g_Rhodomicrobium* (*p* < 0.05). Correlation analysis based on FeRB (Figure 3D) at the genus level and examining factors showed that Fe_o_, ST, and SM were negatively correlated with the abundance of six genera, such as *g_Desulfuromonas* and *g_Sulfitobacter* (*p* < 0.05). Fe-OC was significantly positively correlated with seven genera, such as *g_Shewanella* and *g_Ferrimonas* (*p* < 0.05), and significantly negatively correlated with *g_Rubellimicrobium* (*p* < 0.05).

These results indicated that the FeOB community was significantly affected by the factors examined, while the FeRB community was not significantly affected by them.

### 3.3 Changes of functional genes in Fe-cycling microorganisms and correlation with examining factors

**Figure 4:**
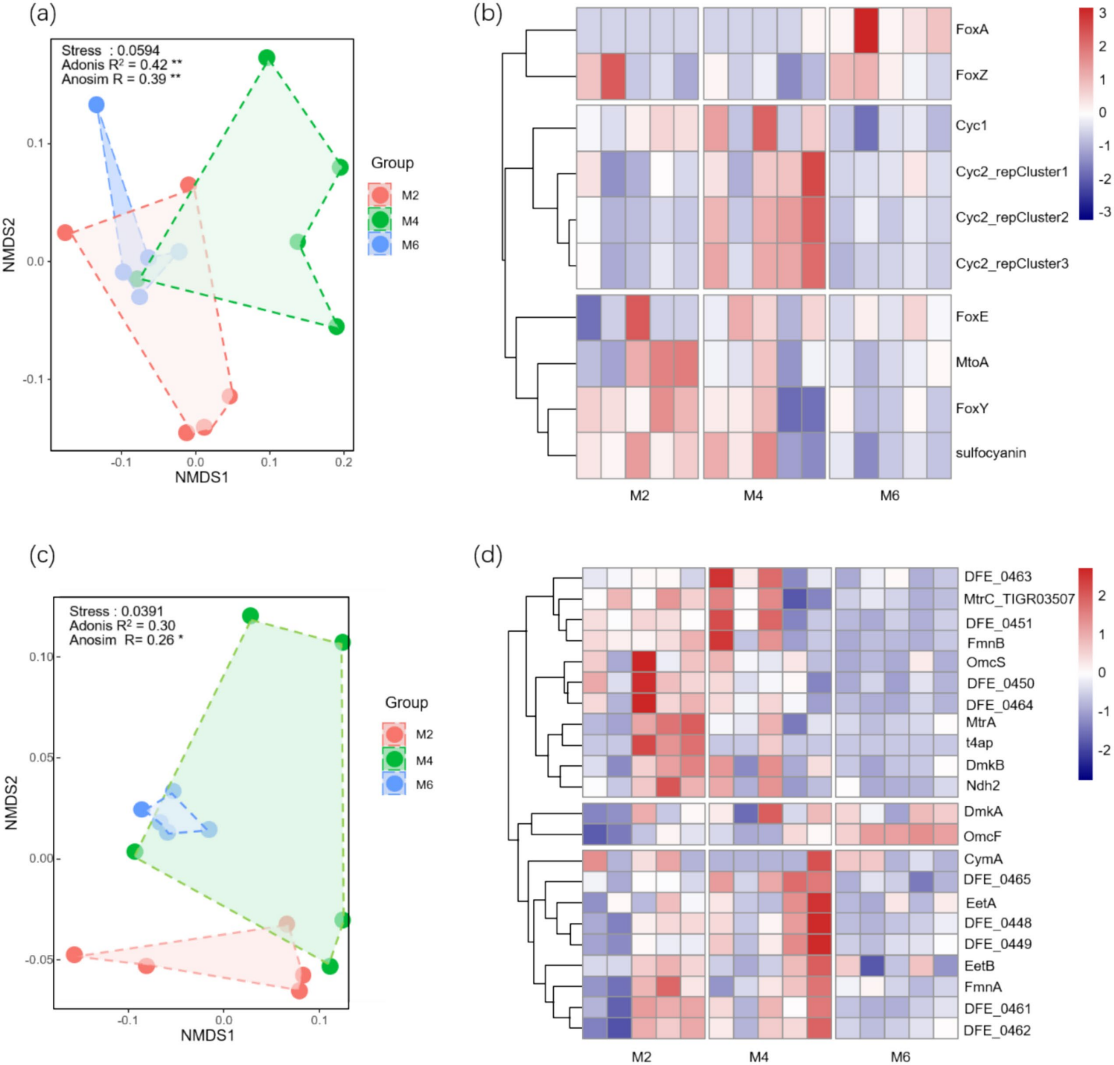
**(a)**Temporal variation characteristics of β diversity in Fe oxidation genes; **(b)** Temporal variation characteristics of Fe oxidation genes composition; **(c)** Temporal variation characteristics of β diversity in Fe reduction genes; **(d)** Temporal variation characteristics of Fe reduction genes composition. * and ** indicate significance at the 0.05 and 0.01 levels, respectively. The points in NMDS represent samples, and different colors represent the information of the group to which the samples belong. The distance between points in the same group indicates the degree of dispersion of samples. M2, deep freezing period (early February); M4, initial thawing period (early April); M6, complete thawing period (early June).

Adonis substitution multivariate analysis of variance (R^2^ = 0.42, *p* < 0.01) and ANOSIM similarity analysis (R = 0.39, *p* < 0.01) revealed significant differences among Fe oxidation genes (Figure 4A). Adonis substitution multivariate analysis of variance (R^2^ = 0.30, *p* < 0.01) and ANOSIM similarity analysis (R = 0.26, *p* < 0.01) revealed significant differences among the Fe reduction genes (Figure 4C). Clear clustering of Fe oxidation genes was observed when frozen soil was thawed (Figure 4B). Sub 1 contained two kinds of genes, which were higher in the M6 group. Sub2 contained four genes, namely, *Cyc1*, *Cyc2_reCluster1*, *Cyc2_reCluster2*, and *Cyc2_recluster3*, which were higher in the M4 group. Marked clustering of Fe reduction genes was observed (Figure 4D). Sub1 contained 11 types of genes, and sub3 contained 9 types of genes, all of which were relatively abundant in the M2 and M4 groups.

**Figure 5:**
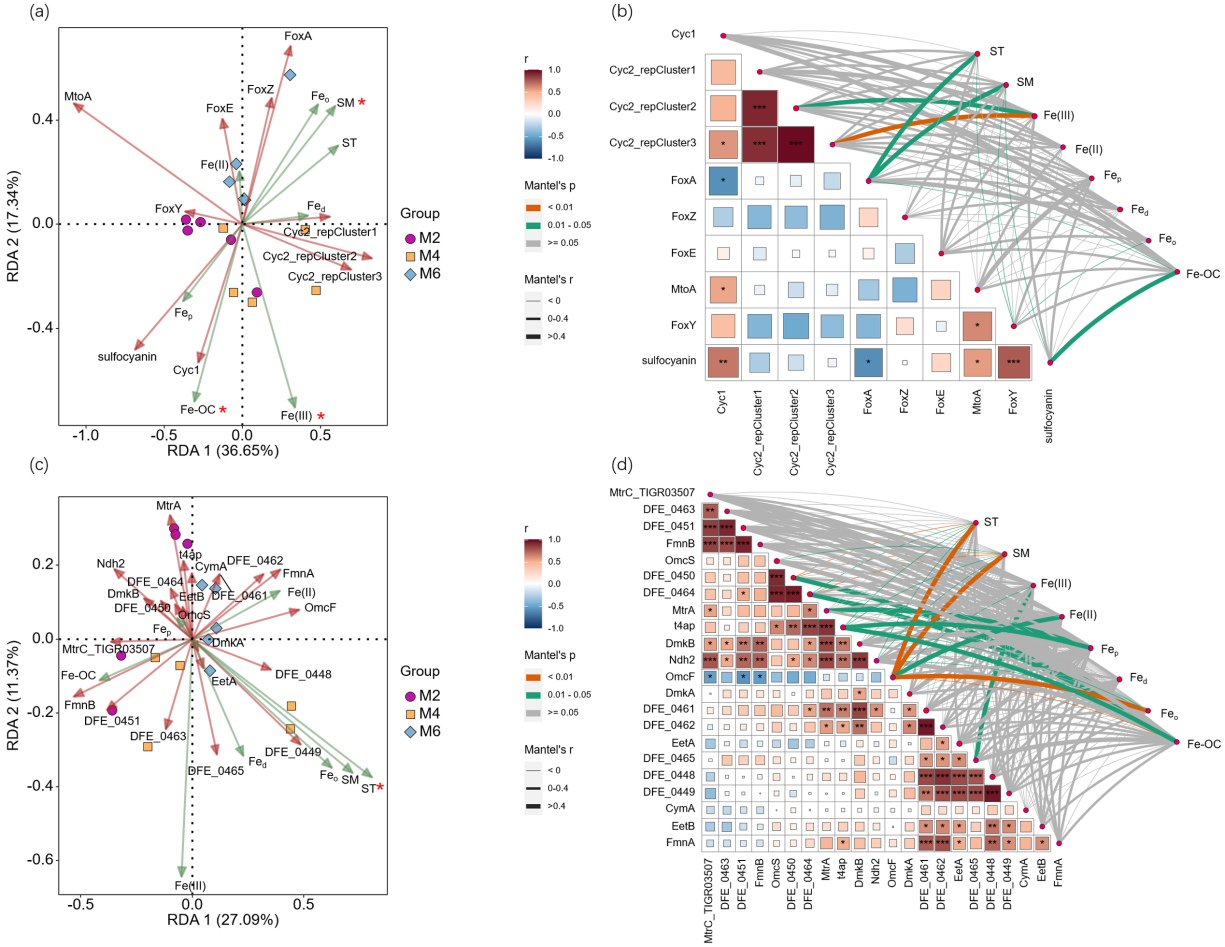
**(a)** RDA analysis was performed with Fe oxidation genes as the response variables and ST, SM, Fe(III), Fe(II), Fe_d_, Fe_o_, Fe_p_, and Fe-OC as the explanatory variables; **(b)** Correlation analysis between Fe oxidation genes and examining factors; **(c)** RDA analysis was performed with Fe reduction genes as the response variables and ST, SM, Fe(III), Fe(II), Fe_d_, Fe_o_, Fe_p_, and Fe-OC as the explanatory variables; **(d)** Correlation analysis between Fe reduction genes and examining factors. *, **, and *** indicate significance at the 0.05, 0.01, and 0.001 levels, respectively. ST, soil temperature; SM, soil moisture; Fed, free Fe oxides; Feo, poorly crystalline Fe oxides; Fep, organically complexed Fe oxides; Fe-OC, Fe-bound organic carbon; M2, deep freezing period (early February); M4, initial thawing period (early April); M6, complete thawing period (early June).

RDA analysis was performed with FeOB or FeRB functional genes as the response variables and ST, SM, Fe(III), Fe(II), Fe_d_, Fe_o_, Fe_p_, and Fe-OC as the explanatory variables. Fe oxidation genes were significantly affected by SM, Fe(III), and Fe-OC (*p* < 0.05; Figure 5A). Fe reduction genes were only significantly affected by ST (*p* < 0.05; Figure 5C). Correlation analysis of Fe oxidation genes and examining factors revealed that ST, SM, and Fe-OC were all significantly correlated with *FoxA* (*p* < 0.05; Figure 5B). ST, SM, and Fe_o_ were negatively correlated with *FoxY* (*p* < 0.05). SM, Fe_o_, and Fe-OC were significantly correlated with *sulfocyanin* (*p* < 0.05). Correlation analysis of Fe reduction genes and examining factors revealed that ST, SM, Fe_o_, Fe-OC, Fe(II), and Fe(III) were significantly correlated with *OmcF* (*p* < 0.05; Figure 5D). Fe-OC was positively correlated with *DFE-0450*, *DFE-0464*, and *t4ap* (*p* < 0.05) and negatively correlated with *OmcF* (*p* < 0.01).

These results indicated that compared with Fe reduction genes, Fe oxidation genes were greatly influenced by the factors examined.

### 3.4 Mechanism of changes in Fe-OC caused by the thawing of seasonal frozen soil

**Figure 6:**
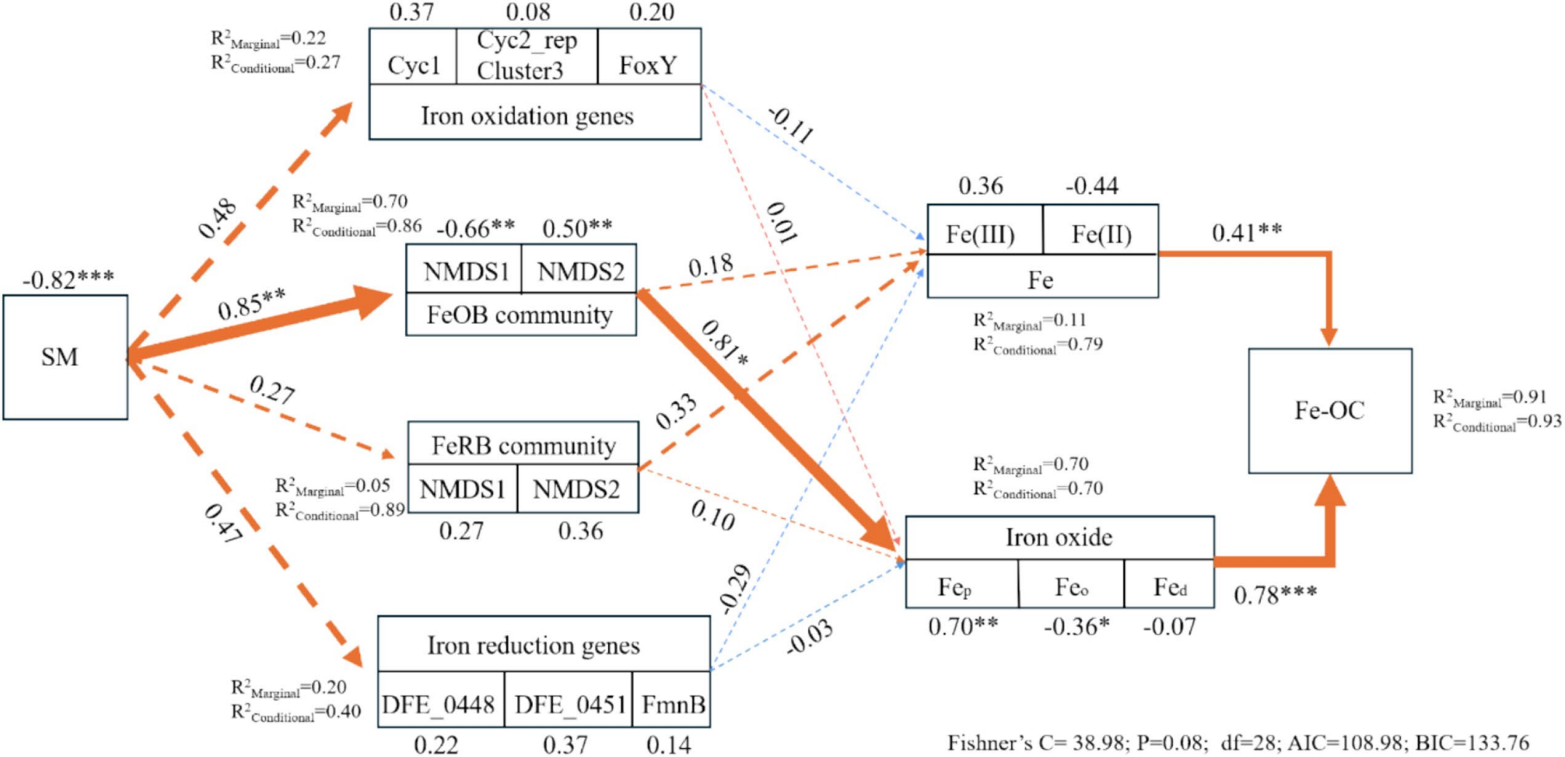
Indirect and direct effects of factors affecting Fe-OC. SM, soil moisture; Fe_d_, free Fe oxides; Fe_o_, poorly crystalline Fe oxides; Fe_p_, organically complexed Fe oxides; Fe-OC, Fe-bound organic carbon. Fe oxidation genes, FeOB, FeRB, Fe reduction genes, Fe, and Fe oxide variables were divided into compound variables. The numbers adjacent to the measured variables are their coefficients with the composite variables. The number adjacent to the arrow is the path coefficient, which is the direct standardized effect. The dashed lines indicate the significance of the relationship. The standardized effect of comprehensive variables on Fe-OC is shown in the marginal and conditional R^2^, which represents the proportion of variance explained by all predictive variables. The relationship between the residual variables that measure the predictor is not shown.

To further reveal the direct and indirect effects of examining factors on changes in Fe-OC (Figure 6), three Fe oxidation and Fe reduction genes that showed the most pronounced changes were used to construct a composite structural equation model. When the random effect of “sampling point” was not considered, Fe oxidation genes, FeOB, FeRB, Fe reduction genes, Fe, and Fe oxides jointly explained the effect of SM on Fe-OC (91%). There was a direct positive effect of Fe phase on Fe-OC, and the effect of Fe oxides was greater. Fe(II), Fe(III), Fe_p_, and Fe_o_ were important factors that directly affected changes in Fe-OC, and the effect of Fe_p_ and Fe_o_ was significant. FeOB had a significant effect on changes in Fe-OC, in addition to a direct regulatory effect. These results indicated that changes in SM due to the thawing of seasonal frozen soil and the change of SM significantly affected the FeOB and Fe oxides (Fe_p_ and Fe_o_), which significantly affected the Fe-OC content.

## 4 Discussion

### 4.1 Effects of seasonal frozen soil thawing on Fe phases

We found that the content of Fe(III) in the subsoil was always higher than that of Fe(II). This is because in the natural environment, Fe(III) is more stable than Fe(II), and it is not easy to be reduced (Patzner et al., 2022). There is always a redox cycle of Fe in soil. The change in soil water content caused by the thawing of seasonal frozen soil will have an impact on soil redox conditions (Joss et al., 2022). The biogeochemical cycle of Fe is intricately linked to the sequestration and mineralization of SOC (Song et al., 2022). The change of soil redox conditions can affect the transformation of Fe(II) and Fe(III), which, in turn, affects the preservation and decomposition of SOC (Widdel et al., 1993). Generally speaking, the oxidation of Fe(II) is beneficial to the fixation of SOC, and the reduction of Fe(III) is beneficial to the mineralization of SOC (Riedel et al., 2013). This was consistent with our results that Fe(II) was negatively correlated with SOC, while Fe(III) was positively correlated with SOC (Figure 6). At the same time, there is a strong correlation between Fe oxides and C storage, and different Fe oxides have different effects on the stability of SOC (Song et al., 2022). We found that the total amount of amorphous Fe oxides (Fe_o_ and Fe_p_) was dominant, which is consistent with previous studies (Duan et al., 2020). Fe_p_ can form a complex with OC. OC retained by Fe_p_ is non-reducible, which makes it accumulate in soil (Coward et al., 2017; Huang et al., 2020); therefore, there was a significant positive correlation between Fe_p_ and Fe-OC. The thawing of frozen soil is beneficial to the dissolution of minerals and drives the mobilization of Fe and C, which increases along the thawing gradient, and this cannot prevent the release of C during thawing (Patzner et al., 2022). The content of Fe-OC decreased with the freezing and thawing cycles, which is consistent with hypothesis 1. During the thawing of swamp soil, Fe-OC may even be completely lost under complete anaerobic reduction (Chen et al., 2020).

### 4.2 Effects of seasonal frozen soil thawing on Fe-cycling microorganisms and functional genes

Due to the high sensitivity and adaptability of microorganisms to the changing environment (Zhao et al., 2024; Chen et al., 2024), we predicted that FeRB should have high environmental sensitivity (hypothesis 2). However, we found the opposite pattern (Figure 3). FeRB tends to have strong environmental adaptability; it can survive and reproduce under extreme conditions such as low temperature and low oxygen and employs a unique survival strategy in the frozen soil environment (Kim et al., 2024). Even if the soil environment changes greatly during the thawing of frozen soil, FeRB can adapt to the new environmental conditions by modifying its physiological mechanisms and metabolic pathways (Sannino et al., 2023). However, the metabolic activity of FeOB depends more on enzymatic reactions. The activity of enzymes is directly affected by temperature (Zuccarini et al., 2023). This was consistent with our findings that FeOB had high environmental sensitivity and was more susceptible to the thawing of frozen soil. FeOB is a kind of aerobic or micro-aerobic microorganism, which takes oxygen as the final electron acceptor. In an oxygen-rich environment, FeOB can rapidly oxidize Fe(II) to form Fe(III) and corresponding Fe oxide precipitates (Rentz et al., 2007). SM also had an important influence on the activity of FeOB. A moderate amount of water can keep the soil moist, which is favorable to the growth and metabolic activities of microorganisms. However, excessive moisture may lead to the decrease of oxygen content in soil and form an anaerobic environment, which is not conducive to the survival and metabolism of FeOB (Emerson et al., 2010). The thawing of frozen soil not only changes the soil aeration but also redistributes the water in the soil (Rodenhizer et al., 2023), and leads to changes in soil redox conditions. The oxidation environment is more beneficial to the generation and reproduction of FeOB. Functional genes are the molecular basis of microbial life activities, and their expression and regulation directly affect the growth, development, and function of microorganisms. As the carriers of functional genes, microorganisms have an important impact on the environment and life activities through their life activities, such as decomposition, synthesis, biodegradation, energy conversion, and metabolism (Liao et al., 2023). Microorganisms adjust the expression of their functional genes according to environmental conditions and maintain life activities. Microorganisms can directly participate in Fe dissolution, redox, and other processes, while functional genes mainly participate in the Fe cycle by regulating the Fe oxidation and Fe reduction processes of microorganisms (Liu Y. et al., 2022). Therefore, in the process of seasonal frozen soil thawing, microorganisms played a leading role in the Fe cycle, Fe oxide transformation, and Fe-OC, rather than functional genes.

### 4.3 Fe-oxidizing microorganisms affect the Fe-OC during the thawing of seasonal frozen soil

FeOB exists widely in nature and plays a key role in the biogeochemical cycle of Fe (Andrews et al., 2013). FeOB obtains energy through the process of biological oxidation of Fe(II). These energies provide power for the growth and reproduction of FeOB and influence the dissolution, migration, and deposition of Fe, thereby affecting the distribution and morphology of Fe in the environment and facilitating its interaction with SOC (Emerson et al., 2010). SOC has a significant affinity for Fe(III), and Fe(III) oxides are mainly combined with SOC (Riedel et al., 2013). FeOB is beneficial to the transformation of Fe(II) to Fe(III) and promotes the preservation of C. FeOB also plays a key role in facilitating the formation of Fe oxides. FeOB can promote the transformation of Fe oxides in soil and convert Fe_o_ into more stable Fe_p_ (Emerson et al., 2010). Fe_p_ is an important non-absorbing storage mechanism of SOC in soil that can promote the stability of SOC because of its irreversibility (Duan et al., 2020). Changes in soil water content are important factors controlling SOC and Fe mineral accumulation (Kramer and Chadwick, 2018). The protective relationship between Fe and SOC is strongly affected by water-sensitive redox kinetics (Buettner et al., 2014); microbial activity is also affected by the SM content (Huang and Hall, 2017). In general, SOC mineralization is positively correlated with SM when SM is low. However, SOC mineralization is negatively correlated with SM when SM exceeds the optimal value. Increases in the SM content inhibit increases in the SOC content caused by soil respiration, which is offset by the release and mineralization of OC combined with Fe oxides (Chen et al., 2020). Therefore, the thawing of seasonal frozen soil had a significant effect on SM, whereas FeOB was more environmentally sensitive and strongly influenced by SM. FeOB affected the transformation of Fe(II) and Fe(III), as well as the transformation of Fe oxide morphology, and ultimately had an impact on Fe-OC content.

## 5 Conclusion

The succession of soil Fe-cycling microorganisms and their decoupling from Fe oxides and Fe-OC were important for exploring the stabilization mechanism of the soil C pool during the thawing of seasonal frozen soil in the alpine grassland of Central Asia. This was the first study to examine the influence of Fe-cycling microorganisms on the Fe phase and Fe-OC in the soil of alpine grassland in Central Asia. During the thawing of frozen soil, the Fe-OC content in the subsoil of alpine grassland in Central Asia decreased significantly. FeOB was more affected by freezing and thawing than FeRB. Seasonal freezing and thawing significantly affected FeOB and Fe oxides (Fe_p_ and Fe_o_) in the subsoil of alpine grassland, which significantly affected the content of Fe-OC. Our findings provide scientific clues for exploring the biogeochemical cycle process in future climate change.

## Data Availability

The datasets presented in this study can be found in online repositories. The names of the repository/repositories and accession number(s) can be found at: https://www.ncbi.nlm.nih.gov/, PRJNA1180693.
